# *ShenLingLan* Influences the Attachment and Migration of Ovarian Cancer Cells Potentially through the GSK3 Pathway

**DOI:** 10.3390/medicines4010010

**Published:** 2017-02-21

**Authors:** Sioned Owen, Fiona Ruge, Yunong Gao, Ying Yang, Jianqing Hou, Jian Chen, Yi Feng, Huiming Zhang, Yong Gao, Hongtao Wang, Cong Wei, Yiling Wu, Wen G. Jiang

**Affiliations:** 1 Cardiff China Medical Research Collaborative, Cardiff University-Peking University Joint Cancer Institute, Henry Wellcome Building, School of Medicine, Cardiff University, Cardiff CF14 4XN, UK; OwenS15@cardiff.ac.uk (S.O.); Ruge@cardiff.ac.uk (F.R.); FengY13@cardiff.ac.uk (Y.F.); ZhangH52@cardiff.ac.uk (H.Z.); 2 Department of Gynaecological Oncology, Peking University Cancer Hospital, Haidian District, Beijing 100142, China; gaoyunong@vip.sina.com; 3 Departments of Gynaecology and Medical Oncology, YuHuangDing Hospital, QingDao University, Yantai 266061, China; hxdany2004@126.com (Y.Y.); hjq63@126.com (J.H.); chenjianyt@163.com (J.C.); 4 Yiling Medical Research Institute, Shijiazhuang 050000, China; gaoyong@yiling.cn (Y.G.); wanghongtao@yiling.cn (H.W.); weicong@yiling.cn (C.W.); 5 State Key Laboratory of Collateral Disease Research and Innovation Medicine, Shijiazhuang 050000, China; 6 Key Disciplines of State Administration of TCM for Collateral Disease, Shijiazhuang 050000, China

**Keywords:** *ShenLingLan*, ovarian cancer, cell migration, GSK3

## Abstract

**Background:** Ovarian cancer presents a major clinical challenge in the UK. Glycogen synthase kinase-3 (GSK-3) has been linked to cancer. This study tested the impact of *ShenLingLan* (*SLDM*) on ovarian cancer cell behaviour and its links to GSK-3. **Methods:** Fresh ovarian tumours (*n* = 52) were collected and processed. Histopathologcial and clinical information were collected and analysed against GSK-3 transcript levels using quantitative PCR (qPCR). Immortalised ovarian cancer cells’ protein alterations in response to *SLDM* were identified using a Kinexus™ protein kinase array. The effects of *SLDM* and a combination of *SLDM* and TWS119 on ovarian cancer cells ability to attach and migrate were evaluated using electrical cell-substrate impedance sensing (ECIS). **Results:** Transcript expression of GSK-3β was significantly increased in ovarian tumours which were poorly differentiated, patients with recurrence and in patients who had died from ovarian cancer. Treating SKOV-3 ovarian cells with *SLDM* reduced GSK-3 expression and GSK-3α (Y279). Treatment with *SLDM* reduced ovarian cancer cells ability to attach and migrate, which was further reduced in the presence of TWS119. **Conclusions:** This study identified a potential mechanism by which *SLDM* may exert anti-metastatic effects. Further work is needed to investigate the in vivo effects *SLDM* has on ovarian tumours.

## 1. Introduction

Ovarian cancer incidence in women within the UK has increased by 17% since the 1970s and it is the fifth most common cancer-related death, though it is the seventh most common cancer type diagnosed [1]. Ovarian cancer is often referred to as the “silent killer” as almost 60% of cases are diagnosed at an advanced stage of the disease (stages III and IV) [2,3]. Tumour metastasis contributes to the poor clinical outcome associated with all cancers, involving several key behaviours; ovarian cancer cells form metastases by migrating through blood vessels as well as through the peritoneal cavity, affecting the peritoneum, urothelium, liver and lungs, which may already have occurred at time of diagnosis and therefore makes the disease harder to treat [4].

Glycogen synthase kinase-3 (GSK-3) has two highly conserved members, *GSK3A* and *GSK3B*, which result in a 51 kDa and 47 kDa protein, respectively [5]. Both have been identified as critical enzymes involved in a variety of cellular processes, some of which are disrupted in cancer, including cell cycle progression, differentiation, migration and survival [6]. The overall roles GSK-3 may play in cancer development and disease progression are complex. Abnormal GSK-3β expression has been observed in a variety of cancers, including pancreatic, colorectal and ovarian [7,8,9]. In contrast, in breast cancer it has been demonstrated that over-expression of GSK-3β may be linked to increased chemo-sensitivity and reduced tumourigenicity [10,11]. Currently, GSK-3β is known to phosphorylate over 40 proteins including at least 12 transcription factors [12]. For example, evidence shows that GSK-3β modulates p53 activity and also affects c-Myc and NFκB activities which can further regulate many other important genes in cell cycle regulation and oncogenesis [13,14].

*ShenLingLan* is a traditional medicine, formulated for treating cancer patients and there are early signs that this medicine benefits these patients. The present study aims to investigate if and how the medicine may work using a series of methods, in a hope to find and eventually provide scientific support for using this medicine in patients. *ShenLingLan* is made of 14 individual herbs, including *Astragalus*, *Ligustrum lucidum*, *Ginseng*, *Ganoderma lucidum*, *Curcuma*, *Gynostemma*, *Atractylodes* (*fried*), *Poria*, *Cordyceps sinensis*, *Xu changqing*, *Eupolyphaga*, *Panax*, *diffusa*, *Scutellaria barbata*, *Divine Comedy* (*fried*). Currently, *ShenLingLan* is used as a formulated capsule, particularly for those who are receiving chemotherapy. Chen and Wu (2006) demonstrated the safety and efficacy of *ShenLingLan* in their clinical trial for chronic hepatitis B and showed that it also affected T-cell function [15].

We recently reported that another Chinese medicine formula, *YangZheng XiaoJi*, which shares a good degree of similarities in terms of herbal composition, has important properties by acting on endothelial cells and cancer cells, together impacting on cancer metastasis [16,17]. The medicine has also been reported to have a more profound effect on the peritoneal metastatic processes in various cancer types including pancreatic, ovarian and gastrointestinal cancers [18,19,20].

In light of these exciting discoveries, we proposed to investigate if *ShenLingLan* may also exert similar actions and, if so, by what mechanisms. The study investigated the direct effects of *ShenLingLan* on ovarian cancer cell behaviour in vitro and investigated the transcript expression profile of GSK-3β in our ovarian cohort.

## 2. Materials and Methods

Materials and cells. Inhibitor to GSK3β, TWS119, was purchased from Tocris (Bristol, UK). Human ovarian cancer, SKOV3, COV504, OVCAR3, A2780 were purchased from LGC Standard/ATCC (Southampton, UK).

*ShenLingLan* extract (*SLDM*). *ShenLingLan* was obtained from Yiling Pharmaceuticals (Shijiazhuang, HeBei, China). It is made of 14 individual herbs, including *Astragalus*, *Ligustrum lucidum*, *Ginseng*, *Ganoderma lucidum*, *Curcuma*, *Gynostemma*, *Atractylodes* (*fried*), *Poria*, *Cordyceps sinensis*, *Xu changqing*, *Eupolyphaga*, *Panax*, *diffusa*, *Scutellaria barbata*, *Divine Comedy* (*fried*). An extract from *ShenLingLan*, named *SLDM* was prepared using a DMSO-based method that was recently described in full [21]. Briefly, *ShenLingLan* formulated powder was added to pure sterile DMSO solution at a fixed weight/volume ratio. The mixture was placed on a rotating wheel (100 rpm) (Labinco BV, Wolf Laboratories, York, UK) for 12 h in a cold room at 4 °C. The preparation was collected and subject to centrifugation at 15,000 *g*, for 20 min at 4 °C. The clear supernatant was carefully collected and pooled, before filtration using filtration filters which had a pore size 0.20 µm (Sartorius Stedim, Sartorius, Epson, Surrey, UK). The extract was diluted in a balanced salt solution (BSS) and standardised by quantifying the optical density of the preparation using a spectrophotometer at 450 nm wavelength (Biotek, Wolf Laboratory). A master preparation which gave 0.25 OD at 450 nm was stored as the master stock and so named as *SLDM* for the subsequent experiments.

### 2.1. Cell Growth Assay

Cancer cells were first seeded in 96-well tissue culture plates and allowed to adhere. The test materials and their combinations were then added to the cells, which were subsequently incubated for a period of 72 h. After removing the culture media, cells were fixed with 4% formalin and stained with 0.5% Crystal violet. After extensive washing, the staining was extracted with 10% acetic acid and plates were read on a multiple channel plate reader at 540 nm (BIO-TEK, Elx800, Wolf Laboratories, York, UK).

### 2.2. Protein Arrays for Detecting Phosphorylation Changes

Cancer cells at 90% confluence in two T75 tissue culture flasks were washed with BSS buffer and then placed into a fresh batch of DMEM supplemented with 5% FCS. After 5 h, treatment was added, again in 5% FCS. After the 5 h period, cells were removed from the flasks with a cell scraper. The cells were pelleted using a centrifuge at 2500 rpm for 5 min. Lysis buffer was added to the cell pellets and placed on a spinning wheel for 1 h at 4 °C. The lysates were then spun at 12,000 g for 10 min at 4 °C. The supernatant were carefully collected and the insolubles discarded. Based on absorbance, the protein concentration in the cell lysates were quantified using linear regression plot in Microsoft Excel and then adjusted to 2 mg/mL. The samples were stored at −20 °C until use. Antibody based protein arrays, namely Kinex™, which has 878 capture antibodies spotted on to each array slide (Kinexus Bioinformatics Ltd., Vancouver, BC, Canada) were used in the present study. The following are the key parameters collected and used for the data analyses: Globally Normalized Signal Intensity—Background corrected intensity values were globally normalised. The Globally Normalized Signal Intensity was calculated by summing the intensities of all the net signal median values for a sample. Z Scores—Z score transformation corrects data internally within a single sample. Z Score Difference—The difference between the observed protein Z scores in samples in comparison. Z Ratios—Divide the Z Score Differences by the SD of all the differences for the comparison. %CFC defines the changes in intensity between control and treatment samples as the percentage change from control using globally normalised data.

### 2.3. Electric Cell-Substrate Impedance Sensing (ECIS)-Based Analyses on Cell Adhesion and Cell Migration

Briefly, 96-well W96E1 microarrays were used on the ECIS Ztheta instrument (Applied Biophysics Ltd., Troy, NJ, USA). Ovarian cancer cells were added to the wells of the array, cell adhesion was monitored by immediate tracking, over a range of frequencies (1000 Hz to 64,000 Hz), using automated modules. The adhesion was analysed using the mathematical modelling methods as previously described. For cellular migration, confluent ovarian cancer monolayers in the arrays were electrically wounded (2000 mA for 20 s each), after which the migration of the cells was immediately tracked, again over a range of frequencies, as the artificial wound closed. All the experiments were conducted in triplicate.

### 2.4. Ovarian Cancer Cohort, Analysis of GSK3Β Transcript

Fresh frozen ovarian tumours were collected immediately after surgery and stored at −80 °C until use. This was conducted under the approval of the local ethics committee (Peking Cancer Hospital Ethics Committee, Ethics Number: 2006021) with consent obtained from the patients. Some of the patients had received chemotherapy or radiation therapy preoperatively. Patient numbers for each group are in Table 1.

### 2.5. Quantitative Analysis of Gene Transcripts in Cells and Tissues

The transcript levels were quantitatively determined by real-time quantitative PCR using StepOne Plus (Applied Biosystem, Warrington, UK). The quantitative detection was based on the Amplifluor™ probe (Intergen Inc, Oxford, UK) method [22] modified from a previously reported method [23]. Cytokeratin-19 (CK19) was used as the internal house-keeping control. Primer details are given in Table 2. The reaction was carried out under the following conditions: 94 °C for 5 min, 96 cycles at 94 °C for 15 s, 55 °C for 35 s and 72 °C for 20 s. The levels of the transcripts were generated using an internal standard that was simultaneously amplified with the samples, and the results are shown in two ways: levels of transcripts based on equivalent amounts of mRNA, and as a target: CK19 ratio [24].

## 3. Results

### 3.1. The Effect of SLDM Treatment on Ovarian Cancer Cell Viabiability

**Figure 1:**
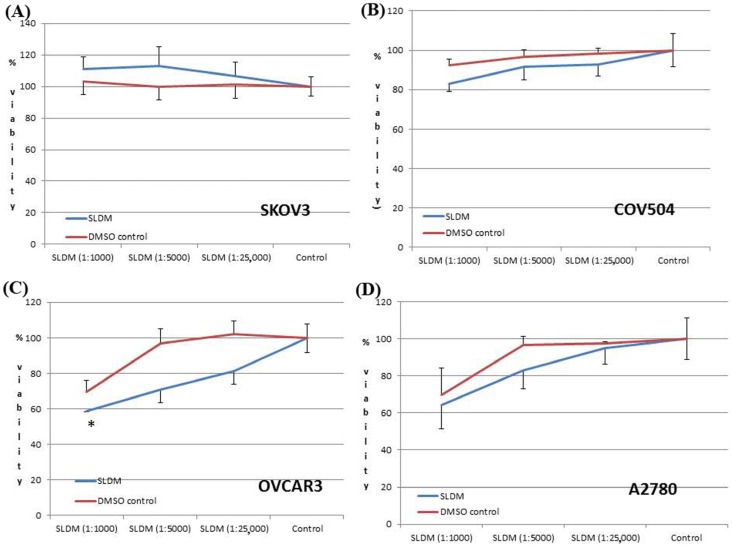
Effects of *SLDM* treatment on ovarian cancer cell viability. At all the concentrations tested, *SLDM* treatment had no significant effect on (**A**) SKOV3, (**B**) COV504 and (**D**) A2780 ovarian cell growth. At the highest concentration (1:1000) tested *SLDM* significantly reduced (**C**) OVCAR-3 cell viability compared to the DMSO control, implying a cytotoxic effect. * *p* < 0.05 vs. control.

*ShenLingLan* had little effect on the viability of three of the ovarian cancer cell lines tested (Figure 1). Over the concentration ranges tested (1:1000 to 1:125,000), SKOV-3, COV-504 and A-2780 cell viability did not significantly differ from the respective DMSO controls (Figure 1A,B,D respectively). When the highest concentration (1:1000) was used on the OVCAR-3 cells, a significant reduction in cell viability was seen compared to the matching DMSO control (*p* = 0.028) (Figure 1C). This significant result implies that at this concentration in this ovarian cancer cell line, *SLDM* treatment may cause toxicity.

### 3.2. SLDM Had a Profound Impact on Cellular Adhesion and Migration of Ovarian Cancer Cells

**Figure 2:**
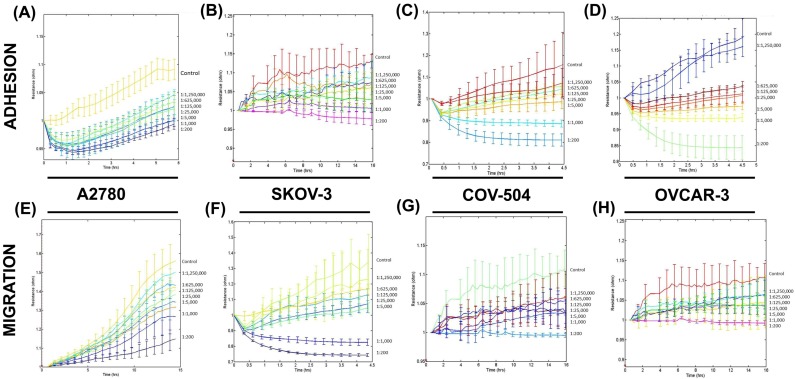
Concentration-dependent inhibition by *SLDM* on the adhesion and migration of ovarian cancer cell lines cells. Increasing concentrations, ranging from 1:200 to 1:1,250,000, of *SLDM* resulted in significantly reduced cell attachment of all four of the ovarian cell lines tested (**A**): A2780; (**B**): SKOV-3; (**C**): COV-504; (**D**): OVCAR-3. A similar trend in all of the ovarian cell migration responses after treatment with *SLDM*; (**E**): A2780; (**F**): SKOV-3; (**G**): COV-504; (**H**): OVCAR-3.

Using the high-throughput screening tool ECIS, it was found that *SLDM* treatment inhibited both ovarian cancer cell adhesion and migration in a concentration-dependent manner (Figure 2). The effects on ovarian cancer cell adhesion were seen in all four of the cell lines tested. In the A2780 and SKOV-3 ovarian cancer cells (Figure 2A,B, respectively), cell attachment was seen at all the concentrations tested; however, the rate of attachment was reduced with increasing concentrations of *SLDM*. In contrast, in the COV-504 and OVCAR-3 cells at the highest concentration tested (1:200), cell attachment was prevented (Figure 2C,D, respectively).

A similar trend was observed for ovarian cancer cell migration when cells were treated with *SLDM*. In all the cell lines tested with increasing concentrations of *SLDM*, ovarian cancer cell migration was reduced (Figure 2D–H, respectively).

### 3.3. Protein Kinase Array Revealed that SLDM Inhibited Phosphorylation of GSK3 in Ovarian Cancer Cells

**Figure 3:**
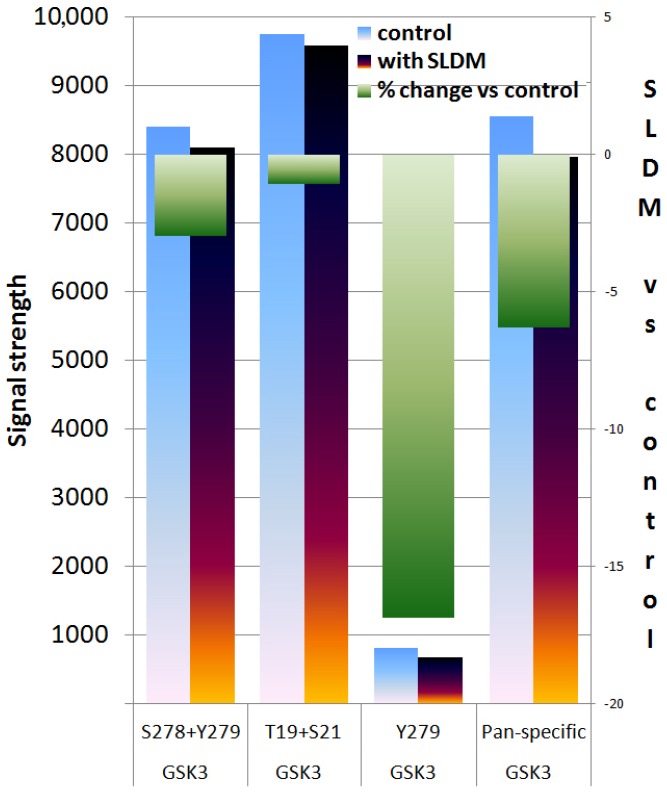
Protein array of GSK3 changes in ovarian cells after treatment with *SLDM*. Signal strength, indicative of expression, for GSK-3 in SKOV-3 cells treated with *SLDM* was reduced when compared to the control. Furthermore, all the phosphorylation sites analysed on the protein array (S278, Y279, T19 and S21) were also reduced in signal strength compared to the untreated control. The green bars indicate the changes in signal strength as a representative of percentage control.

From the protein array analysis, all the GSK-3 signal strengths showed a reduction in the SKOV-3 ovarian samples treated with *SLDM* when compared to the untreated control. This change in signal strength was evident in all of the phosphorylation sites analysed, but particularly at the Y279 site when presented as a percentage change to the control (Figure 3).

### 3.4. Impact of SLDM and GSK3 Kinase Inhibitor on Cell Migration

**Figure 4:**
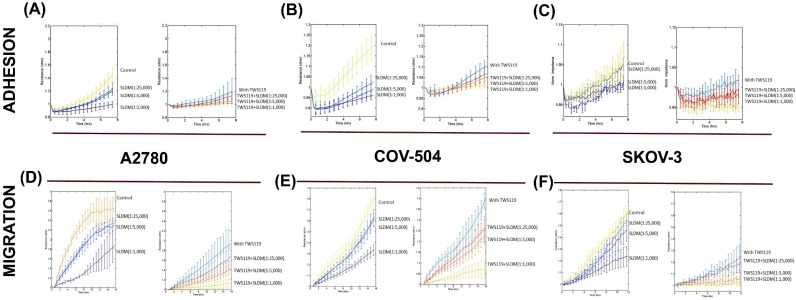
Inhibition of ovarian cell adhesion and migration after treatment with *SLDM* and TWS119. Treatment of A2780, COV504 and SKOV3 cells with a combination of *SLDM* and/or GSK-3β inhibitor TWS119 resulted in decreased cell attachment in all three cell lines ((**A**), (**B**) and (**C**), respectively). Similarly, inhibition of ovarian cell migration after treatment with *SLDM* and TWS119 was also observed in all three cell lines tested, A2780, COV504 and SKOV3, respectively ((**D**), (**E**) and (**F**), respectively).

Based on the protein array data, further ECIS experiments were done to investigate the potential combined effects of a GSK-3β small inhibitor (TWS119) and *SLDM* on ovarian cancer cell attachment and migration (Figure 4). A2780, COV-504 and SKOV3 ovarian cell attachment was significantly decreased by *SLDM* in a dose-dependent manner (from 1:25,000 to 1:1000) and such reduction was enhanced in the presence of TWS119 (Figure 4A–C, respectively). Similar trends were also seen in the migratory patterns of the ovarian cancer cells (Figure 4D–F, respectively). The biggest responses, both in attachment and migration, to the combined treatment of *SLDM* and TWS119 were observed in the SKOV-3 cells (Figure 4C,F, respectively).

### 3.5. GSK3β Expression in Human Ovarian Cancer and the Clinical Implications

**Figure 5:**
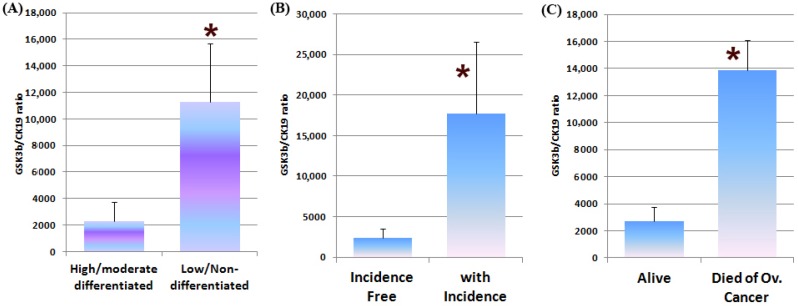
Transcript levels of GSK3β in clinical cohort. When normalised to CK19, GSK3β levels were significantly increased in low and non-differentiated tumours (**A**). Significantly elevated transcript levels were also correlated with recurrence (**B**) and with those patients that had died from ovarian cancer (**C**). * *p* < 0.05.

Expression of GSK3β was linked to a poor clinical outcome. In this relatively small clinical cohort, we found that levels of GSK3β transcripts were significantly higher in patients with ovarian cancer–associated recurrence (*p* < 0.05 vs. incidence free) and in patients who died of ovarian cancer (*p* < 0.05 vs. patients who are alive) (Figure 5B,C, respectively). In addition, poorly differentiated and non-differentiated tumours had significantly higher levels of GSK3β than tumours which were well and moderately differentiated (Figure 5A).

## 4. Discussion

The roles GSK-3 plays in a variety of diseases are extensive due to the vast array of phosphorylation sites and pathways it can regulate. Directional cell migration is a fundamental physiological process which has a wide range of pathways and mediators, one of which is GSK-3 [25]. Within cancer, GSK-3β has been linked to the process of epithelial to mesenchymal transition (EMT). GSK-3β activity is inhibited by the AKT/Wnt and Hedgehog pathways, whilst GSK-3β in turn has been found to phosphorylate several key oncogenic transcription factors as well as serine residues in the EMT marker Snai1 [26].

Here we have demonstrated that *SLDM* inhibits ovarian cell attachment and migration in vitro. Furthermore, in our small clinical cohort, increased levels of GSK-3β were associated with poorly differentiated tumours as well as recurrence and those that had died of the disease. Of further interest was the observation that the combination of *SLDM* and the potent TWS119 GSK-3β inhibitor resulted in a decrease in cellular migration. When both the *SLDM* and TWS119 were combined, reductions in cell migration were noticeable in all three of the ovarian cell lines used. It is of further interest to note that the effects of *SLDM* appeared more significant in the immortalised adenocarcinoma cell line (SKOV-3) rather than in the epithelial-serous carcinoma cell line, COV-504. Ovarian cancer being a heterogeneous disease presents challenges in identifying universally efficacious molecular-targeted therapies. Research by Cao et al. (2006) [7] identified that the ovarian cancer cell line SKOV-3 produces the most active form of GSK-3β. In our study, treating COV-504 cells with TWS-119 did not result in a reduction in cell migration which was observed in the SKOV-3 and A2780 cells. TWS-119, being a selective target inhibitor, may be limited in inhibiting COV-504 cell migration in vitro due to there potentially being less of the necessary drug target.

Furthermore, preliminary protein microarray analysis identified several potential GSK-3β phosphorylation sites which are targeted after ovarian cancer cells are treated with *SLDM.* Literature identifies that phosphorylation of S21 and Y279 inhibits GSK-3α activity [27,28]. GSK-3 is known to interact and influence a wide variety of cellular pathways in a range of complex conditions, including cancer, and one of the main limitations of emerging GSK-3 inhibitors is the potential lack of isoform selectivity for alpha, beta and alpha/beta forms [29]. Cao et al. (2006) previously demonstrated that GSK-3β drove ovarian cell proliferation both in vitro and in vivo through the increased expression of cyclin D1 [7,30]. The identification that *SLDM* targets GSK-3α phosphorylation provides additional interest as this demonstrates that *SLDM* has the potential to target both active proteins’ forms of GSK-3. This inhibitory potential is noteworthy when taken alongside the observations from Fu et al. (2011), who demonstrated in SKOV-3 cells that GSK-3α messenger RNA increased in paclitaxel-resistant cells [31]. In a further study, Fu et al. (2014) also identified that aberrant expression of GSK-3α/β was associated with poor prognosis [32].

Through exploring the potential anticancer properties of *SLDM* and identifying, through Kinexus, phosphorylation events which are potentially induced using this traditional herbal remedy, GSK-3β was one of the molecular targets which was most affected. Both isomers of GSK-3 have been linked to ovarian cancer, alpha in chemoresistance and beta with ovarian cancer cell proliferation. Further work is now required to verify the preliminary protein microarray data and to understand the mechanism of phosphorylation of GSK-3 which is inhibited by *SLDM* throughout a wide range of ovarian cancer cell lines and how these observations in vivo can be used for clinical potential in the future.

## Figures and Tables

**Table 1:** Patients’ clinicopathological information.

Clinical Information	Patient Numbers (n)
**Stage**	
1	3
2	3
3	23
4	6
**Differentiation**	
Low	21
Moderate	1
High	2
**Tumour Subtype**	
Adenocarcinoma	44
Transitional	2
Carcinoma	1
Small Cell Ccarcinoma	1
Nonadenoma	5
**Metastasis**	
No metastasis	36
Metastasis	16
**Incidence**	
Incidence free	25
With incidence	27
**Survival**	
Alive	36
Died	9

**Table 2:** Primer sequences.

Gene Target	Primer Sequence (5′-3′)
**GSK-3β**	
Forward	AACAGGACATTTCACCTCAG
Reverse	ACTGAACCTGACCGTACAGGTGTATACTCCAGCAGACG
**CK-19**	
Forward	AGCCACTACTACACGACCAT
Reverse	ACTGAACCTGACCGTACATCGATCTGCAGGACAATC

ACTGAACCTGACCGTACA—Z sequence for uniprimer probe.
